# Developing an Optimized Protocol for Regeneration and Transformation in Pepper

**DOI:** 10.3390/genes15081018

**Published:** 2024-08-02

**Authors:** Shamsullah Shams, Beenish Naeem, Lingling Ma, Rongxuan Li, Zhenghai Zhang, Yacong Cao, Hailong Yu, Xigang Feng, Yinhui Qiu, Huamao Wu, Lihao Wang

**Affiliations:** 1 State Key Laboratory of Vegetable Biobreeding, Institute of Vegetables & Flowers, Chinese Academy of Agricultural Sciences, Beijing 100081, China; shams.lec18@gmail.com (S.S.); beenishnaveed2020@gmail.com (B.N.); mlingling0224@163.com (L.M.); zhangzhenghai@caas.cn (Z.Z.); caoyacong@caas.cn (Y.C.); yuhailong@caas.cn (H.Y.); fengxigang@caas.cn (X.F.); 2 Sanya National Nanfan Research Institute of the Chinese Academy of Agricultural Sciences, Hainan Yazhou Bay Seed Lab, Sanya 572024, China; lirongx_7@163.com; 3 College of Plant Science and Technology, Beijing University of Agriculture, Beijing 102206, China; 4 Sanming Academy of Agriculture Sciences, Zhuyuan Village, Qiujiang Street Office, Sha County, Sanming 365509, China; qyh3832329@163.com

**Keywords:** *Capsicum annuum*, *in vitro* culture, *Agrobacterium tumefaciens*, explants

## Abstract

*Capsicum annuum* L. is extensively cultivated in subtropical and temperate regions globally, respectively, when grown in a medium with 8 holding significant economic importance. Despite the availability of genome sequences and editing tools, gene editing in peppers is limited by the lack of a stable regeneration and transformation method. This study assessed regeneration and transformation protocols in seven chili pepper varieties, including CM334, Zunla-1, Zhongjiao6 (ZJ6), 0818, 0819, 297, and 348, in order to enhance genetic improvement efforts. Several explants, media compositions, and hormonal combinations were systematically evaluated to optimize the *in vitro* regeneration process across different chili pepper varieties. The optimal concentrations for shoot formation, shoot elongation, and rooting in regeneration experiments were determined as 5 mg/L of 6-Benzylaminopurine (BAP) with 5 mg/L of silver nitrate (AgNO_3_), 0.5 mg/L of Gibberellic acid (GA_3_), and 1 mg/L of Indole-3-butyric acid (IBA), respectively. The highest regeneration rate of 41% was observed from CM334 cotyledon explants. Transformation optimization established 300 mg/L of cefotaxime for bacterial control, with a 72-h co-cultivation period at OD_600_ = 0.1. This study optimizes the protocols for chili pepper regeneration and transformation, thereby contributing to genetic improvement efforts.

## 1. Introduction

Chili is a species of the *Solanaceae* family, which is distributed from central Mexico (cultural region of Mesoamerica) and broad regions of Central and South America. The genus *Capsicum* is composed of 30 species, among them, the domesticated (*C. annuum*, *C. baccatum*, *C. chinense*, *C. frutescens*, and *C. pubescens*), which originated from Mesoamerica (Mexico and Central America) to the Andean region (Ecuador, Bolivia, Chile and Peru), which has contrasting soil, climate, and altitude conditions. The chili pepper (*C. annuum* L.), also referred to as chili or hot pepper, is a widely recognized crop known by various names across the world, including *Capsicum*, Paprika, Pimento, hot pepper, Red pepper, Bell pepper, and Rocoto [1,2]. From a nutritional standpoint, chili fruits are a rich source of carbohydrates, proteins, minerals, ascorbic acid, and vitamins C, A, E, and Capsaicinoids [3,4]. Members of the *Capsicum* family have been observed to exhibit recalcitrance in terms of differentiation and plant regeneration *in vitro* [5,6,7].

Plant regeneration is a crucial aspect of plant tissue culture, relying on the principle of totipotency, which allows plant cells to develop into whole organisms. This process can occur through two main pathways, organogenesis and somatic embryogenesis. Tissue culture techniques offer an efficient method for rapid plant propagation, enabling the production of large numbers of plants within a limited space and without external disturbances [8,9,10]. Among the *Capsicum* species studied, *C. baccatum* exhibited the highest regeneration response, followed by *C. annuum*, while *C. chinense* demonstrated limited regeneration potential under the tested conditions. Various explant types, such as hypocotyls, leaf disks, cotyledons, and cotyledonary petioles, have been evaluated for their regeneration capacity [5,11,12,13]. In different studies, hypocotyl [5] or cotyledon [11,12] explants typically yielded the best shoot regeneration results in *Capsicum* species. Specifically, three different types of explants (hypocotyl, cotyledon, and cotyledonary petiole) were tested, and it was found that the highest number of shoots per explant was obtained from cotyledonary petioles [13].

Plant regeneration in *Capsicum* species is influenced by various factors, including the explant type, plant age, and the concentrations of plant growth regulators in the culture medium [14]. Optimizing these factors is crucial for achieving efficient regeneration through organogenesis or somatic embryogenesis pathways. Moreover, the age of the plant material also influences regeneration efficiency. Previous studies investigated the effect of plant age on callus formation in *Capsicum* leaf explants. They observed that callus formation occurred only in leaf explants obtained from 30-day-old plants when cultured on a Murashige and Skoog (MS) medium supplemented with 2 mg/L of 2,4-D and 1.86 mg/L of 6-Benzylaminopurine (BAP) [13,15].

Optimizing the concentrations of plant growth regulators in the culture medium is crucial for successful regeneration. Swamy et al. studied five varieties of *C. annuum* and found that an MS medium containing 2.0 mg/L of BAP and 0.1 mg/L of 1-Naphthaleneacetic acid (NAA) was most effective for callus formation and shoot regeneration. However, for the green variety, better results were obtained with 3.0 mg/L of BAP and 0.5 mg/L of Indole-3-Acetic Acid (IAA). Another study on *C. annuum* cotyledon explants reported that an MS medium supplemented with 4 mg/L of Thidiazuron (TDZ) yielded the best regeneration response, while shoot elongation and root development were favored by an MS medium with 2 mg/L of Gibberellic acid (GA_3_) and 1 mg/L of IAA. The highest shoot regeneration percentage (80.95%) was achieved on an MS medium with 6.0 mg/L of BA and 0.3 mg/L of IAA [16,17].

Genetic transformation of plants represents a significant advancement in modern science, providing valuable insights into plant biology and revolutionizing crop improvement and commercial farming. Standardizing effective plant transformation methods across species is essential for converting plant molecular biology discoveries into crop improvement. One common approach for achieving genetic improvement is through *Agrobacterium tumefaciens*, which transfers a plasmid fragment containing the gene of interest (known as T-DNA) into host cells. Subsequently, transgenic plants are regenerated using cell and tissue culture techniques [10,18,19]. To determine the suitable strain for pepper transformation, the researchers examined three *A. tumefaciens* strains, AGL1, EHA101, and GV3101, and found that the GV3101 induced the highest number of calli in the cultivar Dempsey [20,21]. Cefotaxime salt has been reported to be effective in controlling bacterial contamination during the screening and bud elongation stages [22]. The transformation efficiencies of two plasmid vectors (CaIA and pRB95) were reported as 20% with CaIA (resulting in 10 transplastomic lines in 50 bombarded plates) and 2% with heterologous pRB95 (resulting in 1 transplastomic line in 50 bombarded plates) [23]. The transformation efficiencies of 0.6% and 1% obtained with two challenging genotypes of sweet pepper are, on average, higher than the 0.03–0.6% recorded for the most responsive genotypes of *C. annuum* [24].

Regeneration and transformation are crucial techniques in plant biotechnology that play a significant role in the improvement of crop traits, such as disease resistance, abiotic stress tolerance, and enhanced nutritional contents. The processes of regeneration and transformation aim to produce genetically modified plants with desired traits for agricultural applications. The specific objectives of this study were to establish a stable and efficient regeneration protocol, as well as to develop a transformation protocol for chili pepper.

## 2. Materials and Methods

### 2.1. Establishment of In Vitro Culture

Seeds of seven chili pepper (*C. annuum* L.) varieties, namely CM334, Zunla-1, ZJ6, 0818, 0819, 297, and 348, were obtained from the Solanaceous Crops Department Laboratory at the Institute of Vegetables and Flowers, Chinese Academy of Agricultural Sciences (CAAS), China. The seeds were thoroughly sterilized to ensure a clean and sterile environment for germination. Firstly, seeds were washed 3 times with sterile distilled water (ddH_2_O) to remove any surface contaminants then the seeds were surface sterilized with 75% ethanol for 1 min followed by 3 rinses with ddH_2_O. Subsequently, the seeds were treated with a 1% Sodium hypochlorite (NaClO) (Sinopharm Chemical Reagent Co., Ltd., Shanghai, China) solution for 30 min with continuous shaking to achieve thorough sterilization. After the treatment, the seeds were rinsed at least five times with sterile distilled water to remove any residual sterilizing agents. After that, sterilized chili pepper seeds were placed on sterile filter paper within a laminar flow hood to maintain a clean and sterile environment conducive to successful germination.

Finally, these sterilized seeds were carefully placed on an MS basal medium with vitamins (PhytoTech Labs, Inc., Lenexa, KS, USA), following the specific arrangement detailed in Table 1 [25]. All seeds were transferred to a designated culture room with a temperature of 26 ± 2 °C and a 16 h photoperiod, to provide optimal conditions for germination. The cotyledons and hypocotyl explants were obtained from 2-to-3-week-old seedlings, while leaf explants were used from 3-week-old plants [26].

#### Regeneration

The process of callus induction involved the culture of three types of explants (cotyledons, hypocotyls, and roots) on an MS medium (4.43 g/L MS + 30 g/L Sucrose (Sinopharm Chemical Reagent Co., Ltd., Shanghai, China) + 8 g/L Agar (Sinopharm Chemical Reagent Co., Ltd., Shanghai, China), pH 5.8) supplemented with varying concentrations of BAP in combination with or without ZR.

The explants were cultured on an MS medium supplemented with varying concentrations of BAP (Beijing Solaibao Technology Co., Ltd., Beijing, China) and TDZ (Beijing Solaibao Technology Co., Ltd., Beijing, China), in combination with IAA (Beijing Solaibao Technology Co., Ltd., Beijing, China), NAA (Beijing Solaibao Technology Co., Ltd., Beijing, China), and with or without the addition of AgNO_3_ (Sinopharm Chemical Reagent Co., Ltd., Shanghai, China), to facilitate shoot development. The experimental design, as outlined in Table 1 and Table 2 included distinct treatment groups representing different combinations of BAP, TDZ, and AgNO_3_, aimed at optimizing shoot induction efficiency and quality in the regenerated plantlets. Callus induction rate (%) = Total No. of callus induction/Total No. of explants × 100, and shoot formation rate (%) = Total No. of shoots formation/Total No. of callus induction × 100.

The impact of GA_3_ (Beijing Solaibao Technology Co., Ltd., Beijing, China) on shoot elongation was investigated by comparing various concentrations of GA_3_, as detailed in Table 1. The application of GA_3_, a potent growth regulator known for its role in promoting stem elongation and cellular expansion, aimed to enhance the efficiency and vigor of shoot elongation in chili pepper tissue culture. Average shoot length (cm) = Total No. of shoot length (cm)/Total No. of shoots.

The rooting process was carefully optimized by supplementing the MS medium with Indole-3-butyric acid (IBA) (Beijing Solaibao Technology Co., Ltd., Beijing, China) at a concentration of 1 mg/L to stimulate root development. After shoot elongation, shoots with a length of 2–3 cm were transferred to the rooting medium containing the specified concentration of IBA, initiating the rooting process and promoting the differentiation of adventitious roots from the basal region of the plantlets. Root induction rate (%) = Total No. of shoots with roots/total No. of shoots × 100. For scaling, we used ImageJ 1.8.0 software [27].

### 2.2. Agrobacterium-Mediated Transformation

Three types of explants (cotyledons, hypocotyls, and leaves) from CM334 and Zunla-1 varieties (*C. annuum* L.) were placed on a pre-culture medium (Table 2) for 24 h. The choice of varieties (CM334 and Zunla-1) was based on their demonstrated higher efficiency and consistency in shoot formation, which are crucial for the success of our transformation experiments. A confirmed *Agrobacterium* colony containing the desired plasmid was selected and inoculated into 10 mL of a Lysogeny Broth (LB) liquid medium supplemented with 50 mg/L of Kanamycin (Beijing Solaibao Technology Co., Ltd., Beijing, China) and 25 mg/L of Rifampicin (Beijing Solaibao Technology Co., Ltd., Beijing, China), and incubated at 28 °C with shaking at 200 r/min for 24 h. An amount of 50 µL of the initial culture was transferred into 5 mL of an LB liquid medium containing 50 mg/L of Kanamycin and 25 mg/L of Rifampicin. Monitoring the optical density (OD_600_) to reach approximately 1.0 and subsequently inoculating into a larger 50 mL culture for 16–18 h, until an OD_600_ of approximately 0.6 is achieved, are essential steps to obtain the desired bacterial concentration for transformation experiments. The bacterial cells were harvested by centrifugation at 4 °C, 6000× *g* for 5 min. The harvested bacterial pellet was washed with 40 mL of MS liquid (4.43 g/L MS + 30 g/L Sucrose, pH 5.8) medium centrifugation at 4 °C and 6000× *g* for 5 min, the washed pellet was re-suspended in 50 mL of MS liquid medium. The explants were submerged in the bacterial suspension adjusted to a total volume of approximately 250 mL to achieve an OD_600_ of around 0.1, and then incubated for 15 to 45 min [5]. After drying the explants on sterile filter paper, they were co-cultivated on a co-cultivation medium supplemented with 20 mg/L of Acetosyringone (Beijing Solaibao Technology Co., Ltd., Beijing, China) for 72 h. After the 72 h co-cultivation period, explants were transferred to selection media as outlined in Table 2 for callus induction containing 30 mg/L of Hygromycin and 300 mg/L of Cefotaxime. Following the callus induction phase, the explants were then transferred to shoot formation media with the above-mentioned antibiotics for shoot induction [26].

#### 2.2.1. Preparation of Agrobacterium Inoculation

**Figure 1:**
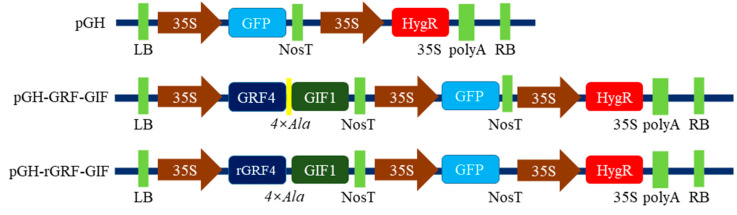
Schematic diagram of the vectors of pGH, pGH-GRF-GIF, and pGH-rGRF-GIF, respectively (Modified from Feng et al. [28]). GFP, Green Fluorescent Protein; GRF4-GIF1, Growth Regulating Factor 4-Growth Interacting Factor 1; HygR, Hygromycin Resistance gene; LB, Left Border of T-DNA; NosT, Nopaline Synthase Terminator; polyA, Polyadenylic acid; RB, Right Border of T-DNA; *4×Ala*, A sequence with four alanine residues.

The process involved transferring three vectors (1. pGH; 2. pGH+GRF-GIF; 3. pGH+rGRF-GIF) [28] provided by Professor Li Yuan from Northwest Agriculture and Forestry University into *A. tumefaciens* strain EHA105 (Shanghai Weidi Biotechnology Co., Ltd., Shanghai, China). The pGH vector is employed to visualize GFP expression under a microscope. The pGH-GRF-GIF vector includes developmental regulator genes (*GRF*-*GIF*) crucial for enhancing regeneration efficiency. Additionally, the pGH-rGRF-GIF vector incorporates a mutated GRF gene aimed at improving the regeneration rate. Further details can be found in previous research [28]. The vectors were introduced into *Agrobacterium* competent cells using the freeze–thaw method, 1 µL of vector was added to 100 µL of competent cells and gently mixed. The mixture was placed on ice for 5 min, then transferred to liquid nitrogen for 5 min, and subsequently heat-shocked in a 37 °C water bath for 5 min. After returning to ice, 700 µL of LB medium (without antibiotics) was added. The cells were incubated at 28 °C on a shaker for 3–4 h, then centrifuged at 6000 rpm for 1 min. After removing 700 µL of supernatant, 50 µL of the remaining cells were plated on LB agar containing 50 mg/L of kanamycin and 25 mg/L of rifampicin. Plates were incubated at 28 °C for 2–3 days to allow bacterial growth. The physical map of gene constructs is explained using the vector diagram (Figure 1).

#### 2.2.2. Molecular Characterization of Transgenic Plants

Transfer and integration of *GFP* and *GGF* (*GGF* is short for the *GRF4* and *GIF1*) genes were analyzed by polymerase chain reaction (PCR) using pairs of designed primers for both genes. Genomic DNA was isolated by the modified CTAB method [29] from fresh, young, and tender leaves of putative transgenic and non-transgenic (Negative control) CM334 and Zunla-1 varieties. The sequences of forward and reverse primers used for *GFP* gene are 5′-CGTAAACGGCCACAAGTTCA-3′ and 5′-CTAGGGAGTCATGGACCGGA-3′, respectively, and for *GGF* genes are 5′-CTCAGGTAGTGGTTGTCGGG-3′ and 5′-TCAGCGACTTGTTCTCATCCAG-3′, respectively. The amplified DNA products separated by agarose gel electrophoresis were documented using the JY04S-3C Gel Documentation Imaging System.

### 2.3. Statistical Analysis

The experiments were conducted using a completely randomized design and data were analyzed with a one-way ANOVA. Mean values were compared through Fisher’s least significant difference (LSD) test, with a significance threshold set at *p* < 0.05. All statistical analyses were performed using Statistic 8.1 software. and GraphPad Prism 8, following the methodology outlined by [30].

## 3. Results

### 3.1. Regeneration Efficiency

#### 3.1.1. Callus Induction

**Figure 2:**
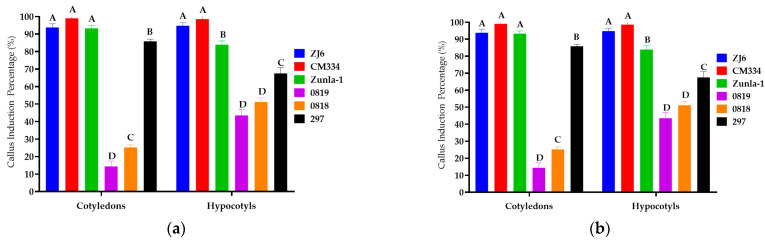

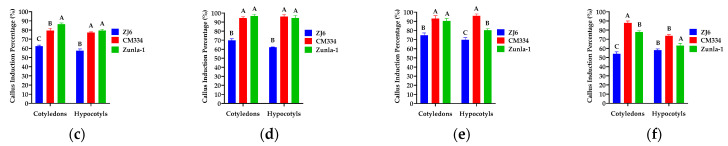
Effect of plant growth regulators (PGRs) and explants on callus induction in different varieties. (**a**) effects of 5 mg/L of BAP + 1 mg/L of IAA + 2 mg/L of ZT (Callus Induction Medium I) on ZJ6, CM334, Zunla-1, 0819, 0818, 297, and 348; (**b**) effect of 5 mg/L of BAP + 1 mg/L of IAA + 2 mg/L of ZR (Callus Induction Medium II) on ZJ6, CM334, Zunla-1, 0819, 0818, 297, and 348; (**c**) effect of 6 mg/L of BAP + 2 mg/L of ZR + 1 mg/L of NAA (Callus Induction Medium III) on ZJ6, CM334, and Zunla-1; (**d**) effect of 2 mg/L of ZR + 1 mg/L of IAA on ZJ6, CM334 and Zunla-1 (Callus Induction Medium IV); (**e**) effect of 4 mg/L of ZR + 5 mg/L of BAP + 1 mg/L of IAA (Callus Induction Medium V) on ZJ6, CM334 and Zunla-1; (**f**) effect of 5 mg/L of BAP + 1 mg/L of IAA (Callus Induction Medium VI) on ZJ6, CM334 and Zunla-1; and explants (cotyledons, hypocotyls, and roots) on callus induction. The ANOVA statistical analysis indicates that there are significant differences in the different letters (A, B, C, D, and E) among the varieties of explants (*p* < 0.05), whereas no significant differences are observed in the same letters among the varieties of explants in treatments.

**Figure 3:**
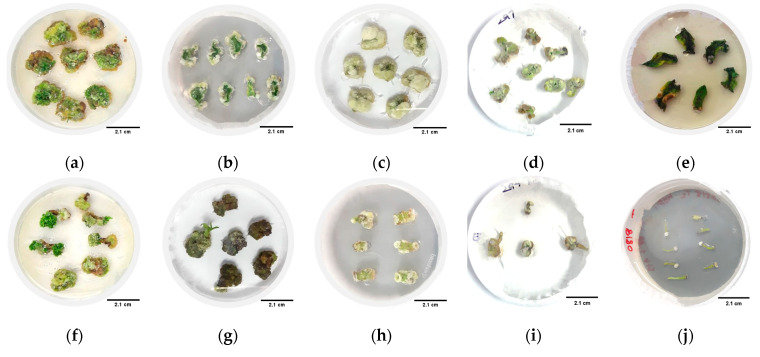
Callus induction in various explants from different varieties. This Figure shows callus formation in CM334, Zunla-1, ZJ6, 297, and 0818 varieties, using cotyledons (**a**–**e**) and hypocotyls (**f**–**j**) as explants, respectively, in callus induction media after 36–48 days. The observed callus formations are displayed on these two specific mediums due to their optimal conditions for callus induction. Scale bars represent 2.1 cm in (**a**–**j**).

Under different concentrations and combinations, the highest callus induction rates were observed for CM334, Zunla-1, ZJ6, 297, 0818, and 0819, with percentages of 99%, 93%, 94%, 86%, 25%, and 14% for cotyledon explants, and 99%, 94%, 95%, 68%, 51%, and 44% for hypocotyl explants, respectively (Figure 2). However, the 348 variety did not exhibit any callus induction. Root explants from all seven varieties did not produce any callus. The study observed variations in callus color among different varieties (Figure 3); callus with lighter or brownish color (Figure 3b–e,h–j) exhibited a lower potential for shoot production. In contrast, dark green callus (Figure 3a,f,g) demonstrated a higher capacity for shoot formation. The dark green callus, often containing more chlorophyll and meristematic regions, indicating a healthier and more differentiated state that supports shoot regeneration. The potential for shoot production varied between different plant varieties, suggesting that genetic factors also play a significant role in callus differentiation and shoot formation.

#### 3.1.2. Shoot Regeneration

**Figure 4:**
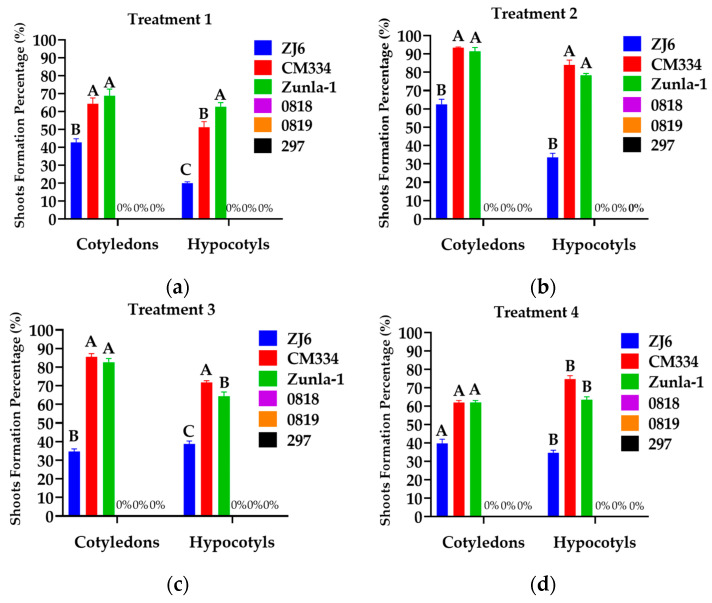
Effect of PGRs and explants on shoot formation in different varieties. (**a**) Effect of 5 mg/L of BAP + 1 mg/L of IAA (Shoot Formation Medium I) on ZJ6, CM334, Zunla-1, 0818, 0819, and 297; (**b**) Effect of 5 mg/L of BAP + 1 mg/L of IAA + 5 mg/L of AgNO_3_ (Shoot Formation Medium II) on ZJ6, CM334, Zunla-1, 0818, 0819, and 297; (**c**) Effect of 6 mg/L of BAP + 1 mg/L of IAA + 5 mg/L of AgNO_3_ (Shoot Formation Medium III) on ZJ6, CM334, Zunla-1, 0818, 0819, and 297; (**d**) Effect of 8 mg/L of BAP + 1 mg/L of IAA + 5 mg/L of AgNO_3_ (Shoot Formation Medium IV) on ZJ6, CM334, Zunla-1, 0818, 0819, and 297; and explants (cotyledons and hypocotyls) on shoot formation. The ANOVA statistical analysis indicates that there are significant differences in the different letters (A, B, and C) among the varieties of explants (*p* < 0.05), whereas no significant differences are observed in the same letters among the varieties of explants in treatments.

**Figure 5:**
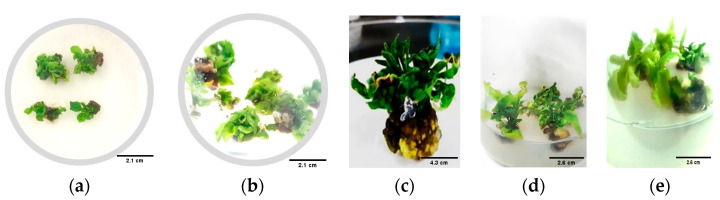
Shows shoot formation in different varieties. Shoot formation initiation of the CM334 (**a**,**b**) occurs after 48–60 days; Zunla-1 (**c**,**d**) after 48–80 days; and ZJ6 (**e**) after 30–60 days, for varieties derived from cotyledons and hypocotyls, respectively, in the shoot formation medium (5 mg/L BAP + 1 mg/L IAA + 5 mg/L AgNO_3_). Scale bars, 2.1 cm in (**a**,**b**), 4.3 cm (**c**), and 2.6 cm (**d**,**e**).

Different concentrations of BAP, along with 1 mg/L of IAA, 0.05 mg/L of NAA, 0.1 mg/L of Inositol and 2 mg/L of ZR, with and without the addition of AgNO_3_, were used for shoot formation. AgNO_3_ (silver nitrate) may play a crucial role in enhancing shoot regeneration in plant tissue culture. Specifically, AgNO_3_ acts as an ethylene inhibitor, which helps to improve shoot regeneration frequency and increase the number of shoots per explant [31,32]. The shoot formation percentage of each cultivar was determined by dividing the total number of shoots formed by the total number of calli and then multiplying by 100. The chili pepper (*C. annuum* L.) varieties CM334, Zunla-1, and ZJ6 exhibited high shoot formation rates of 93%, 91%, and 62% from cotyledon explants and 84%, 78%, and 34% from hypocotyl explants (Figure 4b and Figure 5a,c,e), respectively, when grown in an MS medium supplemented with 5 mg/L of BAP + 1 mg/L of IAA + 5 mg/L of AgNO_3_. Three varieties (0818, 0819, and 297) showed no shoot formation. This outcome highlights the significant genotypic variability exhibited by different pepper varieties in their morphogenic response and competence for shoot formation. CM334 and Zunla-1 exhibited lower shoot formation rates of 62% and 62% from cotyledon explants and 75% and 64% from hypocotyl explants (Figure 4d and Figure 5b,d), respectively, when grown in a medium with 8 mg/L of BAP + 1 mg/L of IAA + 5 mg/L of AgNO_3_.

#### 3.1.3. Shoot Elongation

**Figure 6:**
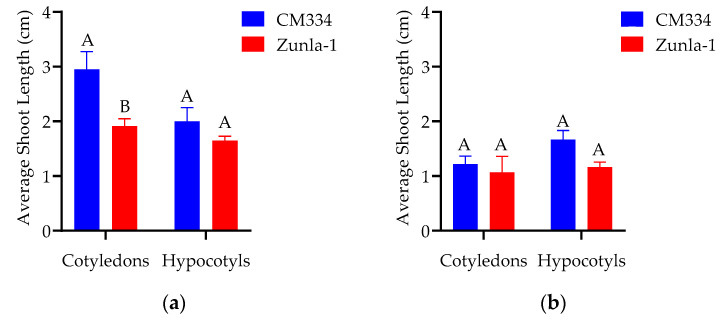
Effect of PGRs and explants on shoot elongation in different varieties. (**a**) The effect of 0.5 mg/L of GA_3_; (**b**) effects of 1 mg/L of GA_3_ on CM334 and Zunla-1 varieties using cotyledon and hypocotyl explants, respectively. This Figure represents the average shoot length for each variety. The ANOVA statistical analysis indicates that there are significant differences in the different letters (A and B) among the varieties of explants (*p* < 0.05), whereas no significant differences are observed in the same letters among the varieties of explants in treatments.

**Figure 7:**
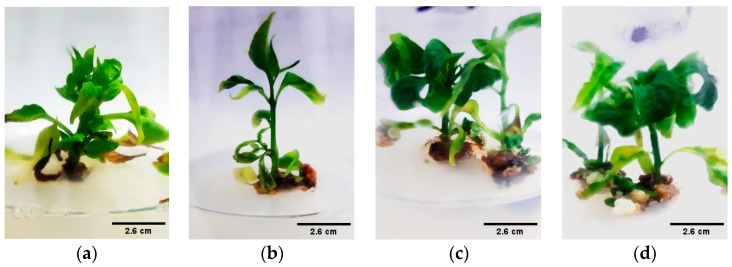
Effect of different PGRs on shoot elongation. This Figure illustrates the shoot lengths of the CM334 (**a**,**b**), and Zunla-1 (**c**,**d**) from cotyledon explants after of 60–90 days of culture in shoot elongation medium supplemented with 0.5 mg/L of GA_3_. Scale bars, 2.6 cm in (**a**–**d**).

For shoot elongation, MS medium was supplemented with sucrose and different concentrations of GA_3_ adjusted to a pH of 5.8. As the seedlings matured, their cells differentiated into shoot and root cells. Different concentrations of GA_3_ were evaluated to identify the optimal level that maximizes the growth of chili pepper explants, as detailed in Table 1. The highest average shoot length of 3 cm was achieved from CM334 cotyledon explants with a GA_3_ concentration of 0.5 mg/L, as illustrated in Figure 6a and Figure 7b. Notably, the experiment observed a decrease in the proliferation rate of explants with the increase in GA_3_ concentration.

#### 3.1.4. Rooting and Acclimatization

**Figure 8:**
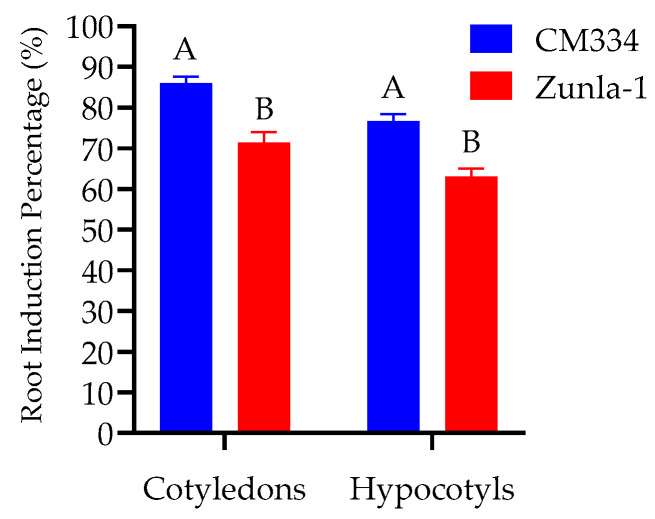
Root induction observed with the application of 1 mg/L of IBA. An amount of 1 mg/L of IBA and explants (cotyledons and hypocotyls) were used on root induction in CM334 and Zunla-1 varieties; The ANOVA statistical analysis indicates that there are significant differences in the different letters (A and B) among the varieties of explants (*p* < 0.05).

**Figure 9:**
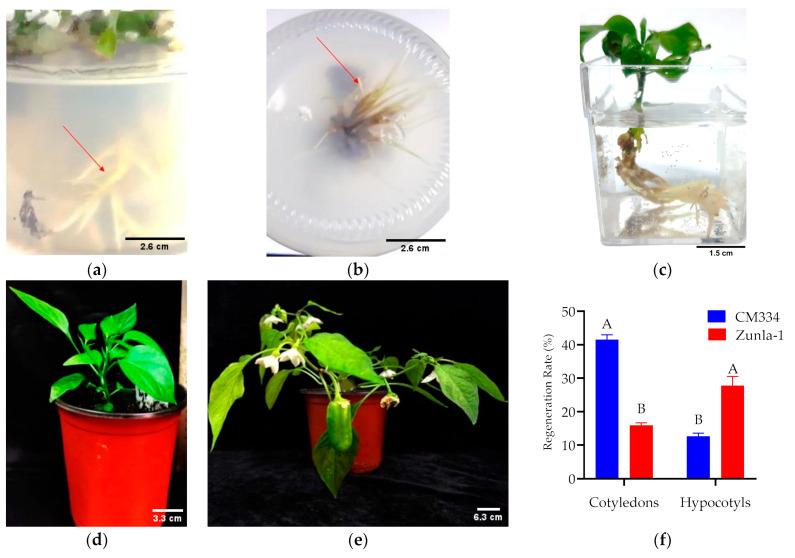
Different stages of the rooting and acclimatization and the regeneration rate. The described roots produced in rooting medium (**a**,**b**) after 20–30 days; Seedlings in tap water for one week (**c**); Seedlings in a Zip pot filled with peat moss soil (**d**,**e**) after 60–120 days. The regeneration rate of cotyledons and hypocotyls of the CM334 and Zunla-1 varieties, respectively (**f**). This Figure represents the regeneration rate on CM334 and Zunla-1 varieties using cotyledon and hypocotyl explants, respectively. ANOVA statistical analysis indicates that there are significant differences in the different letters (A and B) among the varieties of explants (*p* < 0.05). Scale bars, 2.6 cm in (**a**,**b**); 1.5 cm (**c**); 3.3 cm (**d**); and 6.3 cm (**e**).

The induction of root growth was achieved using a concentration of 1 mg/L of IBA (Figure 8), resulting in optimal outcomes. Root emergence from the regenerated shoots was observed within 15–20 days after transferring them to the rooting medium (Figure 9a,b). Subsequently, regenerated shoots with roots from the rooting medium were rinsed with tap water and then soaked in small containers containing water for a week. These containers were placed in the tissue culture room under controlled conditions for one week to enhance acclimatization (Figure 9c). After the one-week acclimatization period in the tissue culture room, the regenerated shoots were transferred to plastic pots and then moved to a greenhouse, where they were subjected to controlled conditions for three to four months (Figure 9d,e). The regeneration demonstrated variability among various plant varieties. The highest regeneration rate of 41% was achieved from CM334 cotyledon explants (Figure 9f).

### 3.2. Transformation

**Figure 10:**
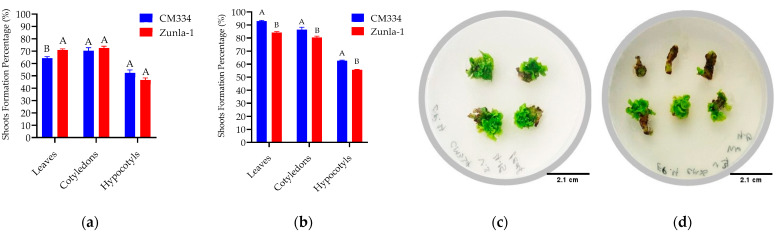
Effect of PGRs and explants on shoot regeneration in the transformation of the different chili pepper varieties. (**a**) Effect of 5 mg/L of BAP on CM334 and Zunla-1 and explants (cotyledons, hypocotyls, and leaves); (**b**) Effect of 4 mg/L of TDZ on CM334 and Zunla-1 and explants (cotyledons, hypocotyls, and leaves); (**c**,**d**) Indicate the shoot formation of the CM334 from leaf and hypocotyl explants, respectively; The ANOVA statistical analysis reveals that different letters (A and B) represent significant differences among the varieties of explants (*p* < 0.05). In contrast, the same letters denote no significant differences among the varieties of explants in treatments.

To develop a stable and effective transformation method for *Agrobacterium*-mediated transformation in chili pepper, we used hypocotyl, cotyledon, and leaf explants from 15-to-18-day-old *in vitro*-grown seedlings of CM334 and Zunla-1, and *A. tumefaciens* strain EHA105. Before infection, the explants were pre-cultured on an MS medium containing 5 mg/L of BAP or 4 mg/L of TDZ to compare the effects of these two concentrations (Table 2). The highest shoot formation rates of 93%, 86%, 84%, and 80% were observed in leaf and cotyledon explants of CM334 and Zunla-1, respectively, when grown in an MS medium containing 4 mg/L of TDZ (Figure 10b).

**Figure 11:**
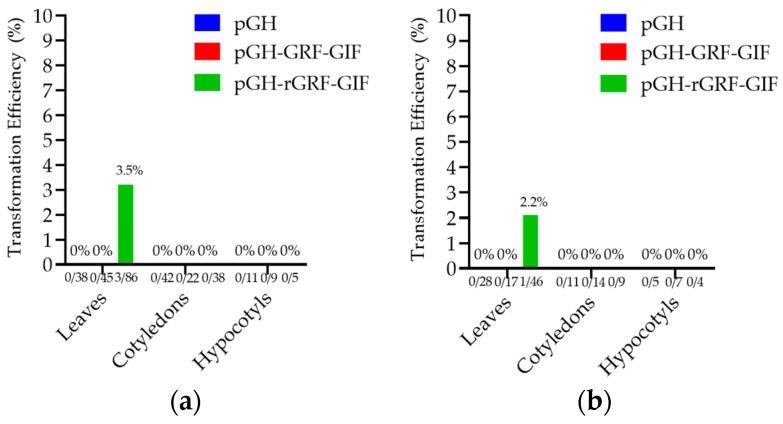
The transformation efficiency. (**a**,**b**) These Figures represent the transformation efficiency of CM334 and Zunla-1 varieties using pGH (Blue), pGH-GRF-GIF (Red), and pGH-rGRF-GIF (Green) vectors, and leaf, cotyledon, and hypocotyl explants, respectively.

Through many experiments, we found that the optimal pre-culture duration, co-cultivation period, and *Agrobacterium* density were determined to be 24, 72 h, and OD_600_ = 0.1, respectively. Following co-cultivation with *Agrobacterium*, the explants were transferred to a selection medium. We carefully optimized the transformation protocol and established suitable concentrations of 300 mg/L of Cefotaxime for effective bacterial control during the transformation process. For a period of two months, the regenerated shoots on the selection media were sub-cultured every 12 days, and the explants underwent the conditions mentioned earlier. The transformation efficiency rates of 3.5% and 2.2% were achieved from CM334 and Zunla-1 leaf explants, respectively (Figure 11a,b).

#### GFP Expression and PCR Analyses

**Figure 12:**
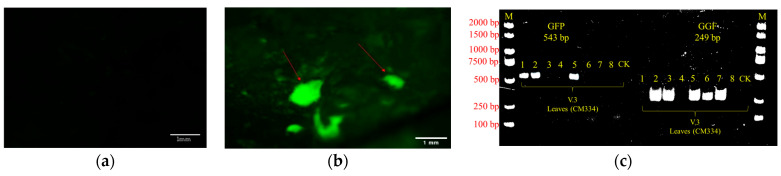
Detection of GFP and *GGF* in transformed plant tissues. (**a**) GFP signals on the leaves of non-transgenic plants (Negative control) using a Leica Microscope (Leica Microsystems Co., Ltd., Wetzlar, Germany). (**b**) GFP signals on the leaves of CM334 observed using a Leica Microscope. The red arrow refers to the GFP positive signal. (**c**) PCR analysis of *GFP* and *GGF* in CM334 leaves. M, Marker; GGF refers to GRF4 and GIF1; Samples 1–8 represent leaves of CM334; CK is the control, that is, ddH_2_O is used as the PCR template; V3, pGH+rGRF-GIF. Scale bars, 1 mm in (**a**,**b**). The PCR fragments observed correspond to the expected sizes of 543 bp for *GFP* and 249 bp for *GGF*.

In the subsequent stages of the plant transformation process, validation of foreign gene integration was conducted by detecting GFP signals and analyzing PCR results from transformed plant tissues. The fluorescence emitted by GFP in transformed plant cells was visually inspected and evaluated, confirming transgene activation in the targeted tissues. This fluorescence was observed and verified using a Leica Microscope, indicating the successful integration of the transgene into the targeted tissues of transformed plant cells. Figure 12b demonstrates this successful integration. Subsequently, PCR was performed to verify the presence of the foreign gene in the transformed plant tissues. The PCR results would typically show specific bands corresponding to the target gene, indicating the presence and successful integration of the foreign gene in the plant’s genome (Figure 12c). In the result of PCR analysis, only leaves 2 and 5 showed positive results for both *GFP* and *GGF* (Figure 12c), indicating that they were more likely transgenic positive leaves. Since we have not verified the presence of the *VirG* gene from *Agrobacterium*, we cannot rule out the possibility that the positive PCR results are originating from residual *Agrobacterium* that may still be present in the plant tissue. Therefore, it is plausible that the other leaf samples (1, 3, 4, 6, 7, and 8), apart from leaves 2 and 5, produced false positives. GFP fluorescence was visible in the *GFP*-positive leaves (2 and 5), confirming the presence of the *GFP* gene. Unfortunately, we did not provide the GFP positive signal picture of leaf sample 5 due to picture quality issues. Conversely, GFP fluorescence was not visible in the *GFP*-negative leaves (1, 3, 4, 6, 7, and 8), which is consistent with the PCR results showing the absence of the *GFP* gene in these samples. In conclusion, based on the PCR analysis results and observation of GFP fluorescence signals, we consider leaf samples 2 and 5 to be the most likely positive transgenic leaves.

## 4. Discussion 

Pepper (*Capsicum* spp.) is an economically important crop within the *Solanaceae* family. The cultivation of chili peppers is believed to have originated around 6000 years ago in eastern-central Mexico [33]. A 2014 study from the University of California Berkeley suggests that chili plants were independently cultivated in various locations across the Americas, including highland Peru and Bolivia, central Mexico, and the Amazon [34].

The use of plant tissue culture or micropropagation, along with recombinant DNA technologies, serves as powerful tools that complement conventional breeding and expedite the improvement of *C. annuum* species [11]. However, the presence of genotype-specific regeneration abilities poses a significant challenge in establishing a standardized regeneration procedure, necessitating specialized regeneration protocols for each cultivar. Among the variables influencing *C. annuum* regeneration, genotypic dependence plays a crucial role, highlighting the importance of optimizing regeneration protocols for specific genotypes or cultivars [35].

Several studies have evaluated the regeneration potential of various *C. annuum* varieties. Four sweet pepper varieties (Hebar, Stryama, Kurtovska kapiya 1619, and Maritsa) were tested, with variety Maritsa exhibiting the highest regeneration response. Similarly, five varieties of *C. annuum* (red, yellow, green, purple, and white) were tested, with the green and red varieties exhibiting the most favorable response. Additionally, eight sweet pepper varieties of *C. annuum* (Dulce Italiano, Agridulce, Toledo, Luesia, Piquillo, Yolo Wonder, Negral, and Lamuyo) were evaluated for regeneration, with Agridulce demonstrating the highest regeneration response [15,36,37]. In the present study, we examined seven *C. annuum* varieties (CM334, Zunla-1, ZJ6, 0818, 0819, 297, and 348) and found that CM334 and Zunla-1 were the most effective for regeneration and transformation.

Several studies have investigated the regeneration of chili pepper plants. *In vitro*, direct organogenesis from hypocotyl explants is the most promising strategy due to its quick and effective organ creation process [12,14,36]. Plant tissue culture is a potential approach for rapid and efficient plant development with several advantages, including the ability to propagate multiple plants in a restricted space without external interference [5,10]. According to these studies, the effectiveness of *in vitro* regeneration, *Agrobacterium* transformation, various explants, and the selection of appropriate plant growth regulator concentrations were evaluated in the present study.

The selection of a suitable explant that responds favorably to regeneration is a crucial factor determining the success of plant propagation through tissue culture. The selection of an appropriate explant that responds to regeneration is a crucial factor in determining the success of plant propagation through tissue culture. Several studies have found that hypocotyls and cotyledons are effective explants for pepper shoot induction and plant regeneration, depending on the variety and study parameters [7,11,25,26,37,38]. In line with these findings, we compered and examined the effects of cotyledons, hypocotyls, roots and leaves on shoot formation during regeneration and transformation processes. Our result indicated that cotyledons and hypocotyls in regeneration, and leaves and cotyledons in the transformation process, were the best explants. Notably, root explants did not yield any shoots, even in callus formation.

Numerous studies have demonstrated favorable results by using specific hormone treatments, such as IAA, BAP, TDZ, and GA_3_, to facilitate shoot formation and elongation in *Capsicum* species [15,16,17]. Previous research has used auxins like IAA and NAA, as well as cytokinins including BAP, TDZ, and ZT, as additional components in the MS medium to investigate their impact on callus induction and chili pepper regeneration [11,39,40]. It has been indicated that the combination of BAP or TDZ with auxins is more effective in inducing shoot formation compared to ZT in chili pepper varieties [17,41]. In contrast, the combination of auxins and cytokinins facilitated shoot development in *C. annuum* L [25]. However, our investigations with CM334 and Zunla-1 revealed that the addition of ZR, along with IAA and NAA, did not support shoot formation. BAP, TDZ, and AgNO_3_ were found to be highly effective in promoting efficient regeneration. Indeed, the most effective approach to stimulate regeneration in both cultivars was observed to be a combination of 5 mg/L of BAP or 4 mg/L of TDZ, supplemented with 5 mg/L of AgNO_3_ and 1 mg/L of IAA.

The genetic transformation of plants represents a significant advancement in modern science, providing valuable insights into plant biology and revolutionizing crop improvement and commercial farming. Genetic engineering technologies heavily rely on genomic changes. This involve a series of steps, including selecting a suitable gene, delivering it, integrating it into plant cells, and expressing it, resulting in the development of a complete plant and molecular identification. Among the various DNA delivery techniques available, *A. tumefaciens*-mediated transformation has been widely used to optimize the chili pepper and improve its traits. *A. tumefaciens* enhances its genetics by inserting a plasmid fragment carrying the desired gene, known as T-DNA, into host cells, followed by growing transgenic plants using cell and tissue culture techniques [42,43]. Increased transformation rates have been observed in the cotyledons, leaves, and hypocotyls of *Agrobacterium* strain LBA4404. Several studies have compared the effectiveness of different *A. tumefaciens* strains, includingAGL1, EHA101, and GV3101, to determine the suitable strain for pepper transformation. These studies found that *A. tumefaciens* strain GV3101 induced the highest number of calli in cv. Dempsey [21,26,44,45,46]. In the present study, we used *A. tumefaciens* strain EHA105, which proved to be an effective strain for achieving successful transformation in our experimental conditions.

The density of bacteria in the inoculation media plays a critical role in the success of transformation. Based on experimental findings, an optimal OD_600_ of 0.2–0.5 was determined to be effective, as higher concentrations resulted in only temporary *GUS* expression [26]. Conversely, researchers conducted a comparison between OD_600_ values of 1 and 0.01, reporting that the high mortality rate associated with the infection could be attributed to the plant’s hypersensitive response to bacterial infection [47]. Our study revealed a direct correlation between the duration of bacterial exposure and the mortality rates of the explants, and prolonged infection periods resulted in an increased mortality rate. The experimental data indicated that an *Agrobacterium* culture density corresponding to an OD_600_ of 0.1, with an infection period ranging from 15 to 30 min, represented the optimal parameters. These conditions minimized the detrimental effects on the explants while ensuring efficient bacterial infection and subsequent transformation.

The duration of co-cultivation plays a crucial role in ensuring successful transformation. Prolonging the co-cultivation period may result in the necrosis and death of the explants due to their hypersensitive reaction. Therefore, it is advisable to keep the co-cultivation period as short as possible to achieve the highest transformation frequency. The experimental findings demonstrated the transformation potential of hypocotyl and cotyledonary explants subjected to varying *Agrobacterium* co-cultivation periods of 24, 48, 72, and 96 h. Their findings identified a 72 h co-cultivation duration as the optimal condition for achieving the highest transformation efficiency [17,26]. Corroborating these previous reports, our experimental observations also indicated that a 72 h co-cultivation period was suitable for efficient *Agrobacterium*-mediated transformation in the plant materials under investigation. This research aligns with similar findings in other plant species [48,49], highlighting the enhancement of transformation efficiency by GRF-GIF chimeric proteins. The successful transformation rates reported in this study demonstrate the effective utilization of the GRF-GIF fusion protein with a mutant miRNA target site [28]. Our findings suggest that the pGH-rGRF-GIF construct achieved successful transformation.

Kanamycin is frequently employed in transformation studies. Whilst Cefotaxime and Carbenicilin are routinely used to control *Agrobacterium* due to their broad-spectrum bactericidal action, according to a study, it has been discovered that Cefotaxime salt is highly effective in inhibiting *Agrobacterium* during the screening and bud elongation stages. Amounts of 500 mg/L of Cefotaxime were examined, which control bacteria contamination [22]. In the present study, 300 mg/L of Cefotaxime was used as the suitable concentration for bacteria control.

## 5. Conclusions

The varieties of CM334 and Zunla-1 demonstrated the highest rates of shoot regeneration and transformation. However, four other varieties (0818, 0819, 297, and 348) did not exhibit any shoot formation. Our results demonstrated that high shoot formation rates were achieved when leaves, cotyledons, and hypocotyls were used as explants in an MS basal medium supplemented with 4 mg/L of TDZ, 5 mg/L of BAP, 1 mg/L of IAA, and 5 mg/L of AgNO_3_. However, roots as explants did not yield any shoots. For shoot elongation, a concentration of 0.5 mg/L of GA_3_ was determined to be optimal, while 1 mg/L of IBA was recommended for rooting. The rate of regeneration exhibited significant variability across different *Capsicum* varieties, with the highest regeneration rate (41%) obtained from CM334 cotyledons as explants.

The *Agrobacterium*-mediated transformation protocol was carefully optimized, with the utilization of 300 mg/L of Cefotaxime for bacterial control throughout the transformation process. Co-cultivation of the explants with *Agrobacterium* cells (OD_600_ = 0.1) for a duration of 72 h was found to be suitable for successful transformation. The highest transformation rate of 3.5% was obtained from CM334 leaves using the pGH-rGRF-GIF vector.

## Figures and Tables

**Table 1:** Media compositions used in regeneration experiments.

Compounds	SGM	CIM I	CIM II	CIM III	CIM IV	CIM V	CIM VI	SFM I	SFM II	SFM III	SFM IV	SEM I	SEM II	RM
MS (g/L)	4.43	4.43	4.43	4.43	4.43	4.43	4.43	4.43	4.43	4.43	4.43	4.43	4.43	4.43
Sucrose (g/L)	30	30	30	30	30	30	30	30	30	30	30	30	30	30
Agar (g/L)	8	8	8	8	8	8	8	8	8	8	8	8	8	8
BAP (mg/L)	-	5	5	6	-	5	5	5	5	6	8	5	5	5
IAA (mg/L)	-	1	1	1	1	1	1	1	1	1	1	1	1	-
NAA (mg/L)	-	0.05	0.05	0.05	0.05	0.05	0.05	0.05	0.05	0.05	0.05	0.05	0.05	-
ZR (mg/L)	-	-	2	2	2	4	-	2	2	2	2	2	2	-
ZT (mg/L)	-	2	-	-	-	-	-	-	-	-	-	-	-	-
Inositol (mg/L)	-	-	-	-	-	-	-	0.1	0.1	0.1	0.1	0.1	0.1	-
GA_3_ (mg/L)	-	-	-	-	-	-	-	-	-	-	-	0.5	1	-
AgNO_3_ (mg/L)	-	-	-	-	-	-	-		5	5	5	-	-	-
IBA (mg/L)	-	-	-	-	-	-	-	-	-	-	-	-	-	1

Note: pH of all mentioned media was adjusted at 5.8. Murashige and Skoog (MS); 6-Benzylaminopurine (BAP); Indole-3-Acetic Acid (IAA); Naphthaleneacetic Acid (NAA); Zeatin Riboside (ZR) (PhytoTech Labs, Inc., Lenexa, KS, USA); Zeatin (ZT) (Beijing Solaibao Technology Co., Ltd., Beijing, China); Gibberellic Acid (GA_3_); Silver Nitrate (AgNO_3_); Indole-3-Butyric Acid (IBA). Seed Germination Medium (SGM); Callus Induction Medium (CIM I,…, VI); Shoot Formation Medium (SFM I and II); Shoot Elongation Medium (SEM I and II); Rooting Medium (RM). After 36–48 days, the callus was transferred from the callus induction media to the shoot formation media.

**Table 2:** Media compositions used in transformation experiments.

Compounds	SGM	PCM I	PCM II	CCM I	CCM II	SM I	SM II	SFM I	SFM II
MS (g/L)	4.43	4.43	4.43	4.43	4.43	4.43	4.43	4.43	4.43
Sucrose (g/L)	30	30	30	30	30	30	30	30	30
Agar (g/L)	8	8	8	8	8	8	8	8	8
BAP (mg/L)	-	5	-	5	-	5	-	5	-
TDZ (mg/L)		-	4	-	4	-	4	-	4
IAA (mg/L)	-	1	1	1	1	1	1	1	1
NAA (mg/L)	-	0.05	0.05	0.05	0.05	0.05	0.05	0.05	0.05
ZR (mg/L)	-	2	2	2	2	2	2	2	2
Inositol (mg/L)	-	-	-	-	-	-	-	0.1	0.1
AgNO_3_ (mg/L)	-	-	-	-	-	-	-	5	5
Acetosyringone (mg/L)	-	-	-	20	20	-	-	-	-
Dithiothretol (mg/L)	-	-	-	150	150	-	-	-	-
Hygromycin (mg/L)	-	-	-	-	-	30	30	-	-
Cefotaxime (mg/L)	-	-	-	-	-	300	300	300	300

Note: We compared the effects of 5 mg/L of BAP and 4 mg/L of Thidiazuron (TDZ) on shoot formation in transformation experiments, pH of all mentioned media was adjusted at 5.8. Seeds Germination Medium (SGM); Pre-culture Medium (PCM I and II); Co-cultivation Medium (CCM I and II); Selection Medium (SM I and II) for callus induction; and Shoot Formation Medium (SFM I and II).

## Data Availability

The original contributions presented in the study are included in the article, further inquiries can be directed to the corresponding authors.
